# Influence of Serum Albumin on HbA1c and HbA1c-Defined Glycemic Status: A Retrospective Study

**DOI:** 10.3389/fmed.2021.583093

**Published:** 2021-05-12

**Authors:** Xiaojing Feng, Yanyi Yang, Siqi Zhuang, Yiyuan Fang, Yufeng Dai, Yaoyang Fu, Qian Hu, Qianqin Yuan, Haoneng Tang, Lingli Tang

**Affiliations:** ^1^Department of Laboratory Medicine, The Second Xiangya Hospital, Central South University, Changsha, China; ^2^Health Management Center of the Second Xiangya Hospital, Central South University, Changsha, China

**Keywords:** albumin, glycosylated hemoglobin, fasting plasma glucose, glycemia status, diabetics

## Abstract

**Background:** Glycated hemoglobin (HbA1c) is commonly used in the diagnosis and evaluation of glycemic control in diabetes, and it may be influenced by several non-glycemic and glycemic factors, including albumin. This retrospective study investigated the influence of albumin on HbA1c and HbA1c-defined glycemic status.

**Methods:** The demographic, hematological, and biochemical data were collected for 11,922 patients undergoing routine physical examination. Univariate and multivariate linear regression analyses, stratified analyses and interaction analyses, and multiple logistic regression were conducted to identify the association between albumin and HbA1c in people with different glycemic status.

**Results:** HbA1c levels were inversely associated with serum albumin level (*P* < 0.0001) in all participants. Risk factors leading to the association included age > 45 years, high fasting plasma glucose (≥7.0 mmol/L), and anemia. The negative association between HbA1c and albumin was curved (*P* < 0.0001) and had a threshold effect in the HbA1c-defined diabetic population; the association was significantly stronger when the albumin level fell below 41.4 g/L (β: −0.31, 95% CI: −0.45 to −0.17, *P* < 0.0001). A 2 g/L increase in albumin reduced the odds of HbA1c-defined dysglycemia, diabetes, and poor glycemia control by 12% to 36%, after adjustment for all possible confounders.

**Conclusions:** HbA1c was inversely associated with albumin level in all participants, and the association was significantly stronger in people with diabetes (defined by HbA1c criteria). For diabetic patients with lower albumin level, there was an increased risk of an erroneous HbA1c-based identification and management of glycemic status.

## Introduction

Assessment of glycemic control is an integral component of effective treatment in the management of diabetes. Monitoring of fasting plasma glucose (FPG) and glycated hemoglobin (HbA1c) are the two main measures available to screen diabetes and assess the effectiveness of present therapy (1, 2). Since 2010, the American Diabetes Association has recommended HbA1c as a parameter in the diagnosis of diabetes and the screening of persons at high risk of diabetes. High HbA1c can strongly predict diabetic complications (3, 4) and cardiovascular morbidity in both diabetic and non-diabetic patients (5, 6). The importance of the HbA1c assay in evaluating long-term glycemic control is well-established (7, 8). However, in spite of strong correlation, data for HbA1c and average FPG show considerable scatter (9, 10), and clinical physicians and patients with diabetes occasionally encounter discordance between the implications of FPG and HbA1c. These may confound the status of glucose management and even mislead the choice for further therapeutic regimen (11, 12).

The International Expert Committee has proposed using the HbA1c level to define impaired glycemic status (13). However, due to stringent quality assurance and standardization requirements (14), the definition of glycemic status based on HbA1c has not been widely implemented in clinical practice in China. Furthermore, the utility of HbA1c measurements in clinical practice also is limited by several non-glycemic and glycemic influences on HbA1c (15, 16). A previous study with a cohort comprising 4,158 type-2 diabetic patients showed reliably that plasma albumin concentration was a predictor of HbA1c, independently of FPG and fructosamine (17). Recently, a study found that the glycation status of albumin also influenced hemoglobin glycation (18). Similarly, densitometry revealed that the extent of glycation was higher in the plasma proteins of diabetic-low albumin mice than in the diabetic-high albumin mice. This reasonably indicates that differences in the glycation of plasma proteins could be due to differential exposure of these proteins to glucose caused by variations in albumin levels (19). However, whether the effect of albumin on HbA1c also exists in normoglycemic and pre-diabetic populations has not been clarified. Moreover, the magnitude of the consequences for diagnosis and management of dysglycemic conditions is uncertain, since if there is a correlation between albumin and HbA1c, some patients may be misclassified by standard HbA1c cutoffs. Therefore, it is necessary to investigate the influence of albumin on HbA1c when defining glycemic status.

This retrospective study investigated the association between serum albumin level and HbA1c value and assessed the degree of influence that albumin has when levels exceed the HbA1c thresholds that are commonly employed for diagnosis and management of diabetes and pre-diabetes.

## Materials and Methods

### Study Participants

All the participants had undergone physical examinations from January 2019 to December 2019 at Second Xiangya Hospital of Central South University in China. The ethics committee of Second Xiangya Hospital of Central South University approved this retrospective study (Ethics Reference No: 089/2016). The study adhered to the principles of the Declaration of Helsinki.

The individuals initially enrolled (*n* = 12,876) were all those aged from 18 to 75 years for whom an analytical profile was obtained (i.e., anthropometric, glycemic, and primary metabolic parameters). Individuals with any of the following were excluded: missing information on covariates; erroneous information on covariates; participants with severe infections; individuals were not Chinese; pregnant women; participants with cancers. Finally, 11,922 participants were included for this analysis (Supplementary Figure 1).

Plasma glucose values for diagnosing diabetes, pre-diabetes, and normoglycemia were identified in accordance with the 2019 criteria of the American Diabetes Association (20). Specifically, diabetes, pre-diabetes, and normoglycemia were defined as, respectively, FPG ≥ 7.0 mmol/L or HbA1c ≥ 6.5%, FPG 5.6–6.9 mmol/L or HbA1c 5.7–6.4%, and FPG <5.6 mmol/L or HbA1c <5.7%. In addition, according to the disease characteristics of the Chinese population, participants with hemoglobin <115 g/L (female) or <130 g/L (male) were deemed anemic. Participants with aspartate aminotransferase (AST) ≥ 70 U/L (female) or ≥80 U/L (male) or alanine aminotransferase (ALT) ≥ 80 U/L (female) or ≥100 U/L (male) were considered to have hepatic disease. Participants with creatinine > 133 μmol/L were considered with renal insufficiency. Hyperlipidemia was defined as total cholesterol (TC) ≥ 5.72 mmol/L or triglyceride (TG) ≥ 1.70 mmol/L or high-density lipoprotein cholesterol (HDL-C) <0.9 mmol/L. The parameters of uric acid (UA) necessary for hyperuricemia were ≥357 μmol/L (female) or ≥420 μmol/L (male).

### Laboratory Measurements

Anthropometric data were collected. Body mass index (BMI) was calculated as weight/height^2^ (kg/m^2^). The systolic and diastolic blood pressures (SBP, DBP) were measured in a sitting resting position with a digital sphygmomanometer and standard protocol.

All blood samples were obtained in the morning (8 a.m.) after a 10- to 12-h overnight fast and then centrifuged (3,000 × *g* for 5 min) for serum separation when applicable. The HbA1c and erythrocyte parameters were determined with whole blood, while other parameters were tested with serum. HbA1c levels were determined by high-performance liquid chromatography using Arkray HA-8160 analyzers (Arkray, Japan). The following hematological parameters were determined with Sysmex XN counters (Sysmex, Japan): red cell count (RBC); hemoglobin; hematocrit (HCT); mean corpuscular volume (MCV); mean corpuscular hemoglobin (MCH); and mean corpuscular hemoglobin concentration (MCHC).

Serum levels of FPG were tested using the glucose oxidase/peroxidase method in Abbott C16000 analyzers (Abbott, America). The following hepatic, renal, and lipid parameters were measured using Abbott C16000 analyzers (Abbott, America): AST, ALT, direct bilirubin (DBIL), albumin, total protein (TP), blood urea nitrogen (BUN), creatinine, UA, total cholesterol, TG, low-density lipoprotein cholesterol (LDL-C), and HDL-C. All analyses were performed on the day of collection in the Department of Laboratory Medicine, Second Xiangya Hospital.

### Statistical Analysis

Normally distributed parameters are reported as mean ± standard deviation and non-normally distributed parameters as median and interquartile range (IQR; Q1–Q3). Univariate statistical comparisons of individual demographics between the albumin quintile groups were conducted for continuous variables using a one-way analysis of variance or a Kruskal-Wallis test when non-normal. Categorical variables are described by numbers with percentages and were compared using the chi-squared test.

Univariate and multivariate linear regression analyses were used to evaluate the effects of serum albumin on HbA1c. A sensitivity analysis was conducted and multivariate adjusted models were used to assess confounding variables. The potential confounders entered into the model were based on their significance in the univariate analysis (*P* < 0.10) or clinical implication. In all the models, the serum albumin levels were treated as both continuous variables scaled in 2 g/L increments and categorical variables divided into quintiles. Trend analyses were also conducted by modeling the albumin quintiles as continuous variables. To confirm the association between serum albumin levels and HbA1c, stratified analyses and interaction analyses were further conducted of the following: gender; age (<45 y and ≥45 y); BMI (18.5–25, 25–28, and ≥28 kg/m^2^); FPG (<5.6, 5.6–7.0, and ≥7.0 mmol/L); and comorbidity (hyperlipidemia, anemia, hepatic disease, renal insufficiency, hyperuricemia).

A two-piecewise linear regression model was applied to examine the threshold effect of serum albumin on HbA1c using a smoothing function. The threshold level was determined using trial and error, including selection of turning points along a pre-defined interval and then choosing the turning point that gave the maximum model likelihood. Using multiple logistic regression with and without adjustment for confounders, also investigated was the influence of increasing albumin levels on the odds of exceeding the HbA1c thresholds most commonly employed for diagnosis of pre-diabetes (5.7%), diabetes (6.5%), and as goals in management (7 and 8%).

A *P*-value <0.05 (two-sided) was considered statistically significant for all tests. All analyses were performed using Empower (R) software (www.empowerstats.com, X&Y Solutions, Boston MA; and http://www.R-project.org).

## Results

### Clinical and Laboratory Characteristics of the Study Participants

Among the 11,922 participants, 6,044 (50.70%) were male (Table 1). Their mean age was 48.66 years, and 62.29% of them were older than 45 years. Their mean BMI was 24.25 ± 3.26 kg/m^2^, and overweight or obese people (BMI ≥ 25) accounted for 38.89%. In the subgroups, the proportion of HbA1c-defined diabetic (8.13%), pre-diabetic (34.78%), and normoglycemic populations (57.09%) were different from that of FPG-defined diabetic (4.69%), pre-diabetic (8.98%), and normoglycemic populations (86.34%; *P* < 0.0001). As for comorbidity, hyperlipidemia was present in 42.89% in all participants, whereas hyperuricemia was found in 12.72% and anemia in 4.32%. Very few participants had hepatic disease (1.11%) or renal insufficiency (0.34%).

**Table 1:** Baseline characteristics of the study participants.

**Variable**	**Overall population**	**Variable**	**Overall population**
Subjects, *n*	11,922	Gender, *n* (%)	
Age, y	48.66 ± 11.17	Male	6,044 (50.70)
BMI, kg/m^2^	24.25 ± 3.26	Female	5,878 (49.30)
Pulse, bpm	77.77 ± 11.14	Age, *n* (%)	
SBP, mmHg	124.40 ± 17.10	≤ 45 y	4,496 (37.71)
DBP, mmHg	76.70 ± 11.41	>45 y	7,426 (62.29)
HbA1c, %	5.60 (5.40–5.90)	BMI, *n* (%)	
FPG, mmol/L	4.75 (4.38–5.19)	<25	7,286 (61.11)
TG, mmol/L	1.35 (0.93–2.00)	25–28	3,195 (26.80)
TC, mmol/L	4.88 ± 0.94	≥28	1,441 (12.09)
HDL-C, mmol/L	1.32 ± 0.29	HbA1c-defined glycemic status, *n* (%)	
LDL-C, mmol/L	2.90 ± 0.78	Normoglycemia	6,806 (57.09)
RBC, 10^12^/L	4.81 ± 0.52	Pre-diabetic	4,147 (34.78)
Hemoglobin, g/L	143.12 ± 16.11	Diabetic	969 (8.13)
Hematocrit, %	43.40 ± 4.25	FPG-defined glycemic status, *n* (%)	
MCV, fL	90.50 ± 5.71	Normoglycemia	10,293 (86.34)
MCH, pg	29.84 ± 2.37	Pre-diabetes	1,070 (8.98)
MCHC, g/L	329.45 ± 12.13	Diabetes	559 (4.69)
ALT, U/L	19.00 (13.90–27.90)	Disease prevalence, *n* (%)	
AST, U/L	21.40 (18.20–25.78)	Hyperlipidemia	5,113 (42.89)
TP, g/L	72.47 ± 3.84	Anemia	515 (4.32)
Albumin, g/L	43.58 ± 2.38	Hepatic disease	132 (1.11)
DBIL, U/L	3.30 (2.50–4.20)	Renal insufficiency	40 (0.34)
Creatinine, μmol/L	67.60 (56.80–80.00)	Hyperuricemia	1,516 (12.72)
BUN, mmol/L	4.99 (4.21–5.91)		
UA, μmol/L	311.78 ± 81.26		

*Data are presented as the mean ± standard deviation or Median (IQR: Q1–Q3) for continuous variables and percentage for categorical variables*.

The clinical and biochemical characteristics of the study population by serum albumin-level quintiles are shown in Table 2. Gender and age distribution varied among albumin levels: participants with lower albumin were predominantly female and older (>45 y) while the participants with higher albumin were mainly male and younger (<45 y). Participants in the fifth quintile of serum albumin level had significantly higher SBP, DBP, RBC, hemoglobin, ALT, AST, DBIL, and creatinine compared with those in the first quintile. There was a significant difference in HbA1c level, but not FPG level, among the albumin intervals, and the mean HbA1c levels significantly decreased from 5.86 to 5.67% (*P* < 0.001 for the trend; Supplementary Figure 2), which ranged across the threshold of HbA1c-defined pre-diabetes (5.7%). In addition, participants in the first quintile of serum albumin level had a significantly higher rate of anemia and renal insufficiency compared with those in the fifth quintile of serum albumin level. However, hyperlipidemia, hepatic disease, and hyperuricemia were significantly more prone to occur in people with higher albumin levels. BMI, FPG, MCH, and BUN did not differ across the albumin quintiles.

**Table 2:** Characteristics of the participants by serum albumin level quintiles.

**Characteristic**	**Serum albumin, g/L**					P^*^
	**Q1 (22.7–41.7)**	**Q2 (41.7–43.1)**	**Q3 (43.1–44.2)**	**Q4 (44.2–45.5)**	**Q5 (45.5–53.4)**	
Subjects, *n*	2,280	2,489	2,308	2,379	2,466	
Age, y	53.30 ± 10.43	50.79 ± 10.50	49.30 ± 10.49	47.09 ± 10.86	43.11 ± 10.82	<0.001
Gender, *n* (%)						<0.001
Male	909 (39.87)	1,017 (40.86)	1,123 (48.66)	1,331 (55.95)	1,664 (67.48)	
Female	1,371 (60.13)	1,472 (59.14)	1,185 (51.34)	1,048 (44.05)	802 (32.52)	
BMI, kg/m^2^	24.24 ± 3.20	24.16 ± 3.19	24.32 ± 3.25	24.21 ± 3.27	24.32 ± 3.38	0.327
Pulse, bpm	76.69 ± 11.29	76.97 ± 10.93	77.16 ± 10.34	77.99 ± 10.94	79.93 ± 11.82	<0.001
SBP, mmHg	123.53 ± 17.72	123.67 ± 17.17	124.44 ± 17.08	124.58 ± 17.06	125.71 ± 16.41	<0.001
DBP, mmHg	75.20 ± 11.22	75.90 ± 11.58	76.62 ± 11.36	77.26 ± 11.28	78.45 ± 11.33	<0.001
HbA1c, %	5.70 (5.40–5.90)	5.60 (5.40–5.90)	5.60 (5.40–5.90)	5.50 (5.40–5.80)	5.50 (5.30–5.80)	<0.001
FPG, mmol/L	4.72 (4.37–5.13)	4.75 (4.37–5.18)	4.77 (4.40–5.21)	4.74 (4.37–5.20)	4.76 (4.39–5.21)	0.854
TG, mmol/L	1.24 (0.89–1.72)	1.27 (0.91–1.86)	1.33 (0.93–2.00)	1.40 (0.95–2.10)	1.50 (1.03–2.33)	<0.001
TC, mmol/L	4.71 ± 0.93	4.88 ± 0.93	4.90 ± 0.91	4.94 ± 0.94	4.97 ± 0.96	<0.001
HDL-C, mmol/L	1.30 ± 0.29	1.33 ± 0.28	1.33 ± 0.29	1.32 ± 0.30	1.30 ± 0.30	<0.001
LDL-C, mmol/L	2.81 ± 0.78	2.92 ± 0.79	2.91 ± 0.77	2.94 ± 0.78	2.94 ± 0.78	<0.001
RBC, 10^12^/L	4.61 ± 0.49	4.71 ± 0.49	4.80 ± 0.50	4.88 ± 0.52	5.03 ± 0.52	<0.001
Hemoglobin, g/L	136.87 ± 15.60	139.94 ± 15.34	143.27 ± 15.53	145.25 ± 15.57	149.89 ± 15.36	<0.001
Hematocrit, %	41.80 ± 4.23	42.59 ± 4.03	43.42 ± 4.16	43.95 ± 4.04	45.13 ± 4.01	<0.001
MCV, fL	90.90 ± 5.83	90.68 ± 5.77	90.59 ± 5.47	90.34 ± 5.86	89.99 ± 5.59	<0.001
MCH, pg	29.77 ± 2.41	29.80 ± 2.40	29.86 ± 2.31	29.85 ± 2.42	29.89 ± 2.31	0.409
MCHC, g/L	327.18 ± 11.87	328.29 ± 11.85	329.61 ± 11.74	330.16 ± 12.55	331.90 ± 12.07	<0.001
ALT, U/L	17.20 (13.00–24.00)	17.60 (13.30–24.70)	18.90 (13.70–27.20)	20.00 (14.60–9.70)	22.70(15.50–33.80)	<0.001
AST, U/L	20.70 (17.60–24.72)	20.70 (17.80–24.60)	21.10 (18.20–25.50)	21.90 (18.70–26.30)	22.30 (18.90–27.00)	<0.001
TP, g/L	69.70 ± 3.85	71.34 ± 3.23	72.31 ± 3.28	73.56 ± 3.10	75.26 ± 3.24	<0.001
Albumin, g/L	40.21 ± 1.57	42.39 ± 0.40	43.60 ± 0.31	44.77 ± 0.37	46.75 ± 1.11	<0.001
DBIL, U/L	3.00 (2.30–3.90)	3.20 (2.50–4.00)	3.20 (2.50–4.20)	3.50 (2.70–4.40)	3.60 (2.80–4.70)	<0.001
Creatinine, μmol/L	63.40 (55.10–77.10)	64.10 (55.30–77.10)	67.00 (56.50–80.00)	69.50 (57.95–81.00)	73.80 (60.50–83.07)	<0.001
BUN, mmol/L	5.00 (4.17–6.00)	4.97 (4.19–5.89)	4.98 (4.22–5.95)	4.99 (4.21–5.85)	4.99 (4.23–5.87)	0.921
UA, μmol/L	295.24 ± 78.87	298.55 ± 76.34	310.09 ± 80.74	318.90 ± 79.83	335.13 ± 83.84	<0.001
Age, *n* (%)						<0.001
≤ 45 y	511 (22.41)	743 (29.85)	807 (34.97)	1,003 (42.16)	1,432 (58.07)	
>45 y	1,769 (77.59)	1,746 (70.15)	1,501 (65.03)	1,376 (57.84)	1,034 (41.93)	
BMI, *n* (%)						0.03
<25	1,426 (62.54)	1,566 (62.92)	1,422 (61.61)	1,433 (60.24)	1,439 (58.35)	
25–28	585 (25.66)	645 (25.91)	594 (25.74)	665 (27.95)	706 (28.63)	
≥28	269 (11.80)	278 (11.17)	292 (12.65)	281 (11.81)	321 (13.02)	
HbA1c-defined glycemic status, *n* (%)						<0.001
Normoglycemia	1,127 (49.43)	1,334 (53.60)	1,307 (56.63)	1,447 (60.82)	1,591 (64.52)	
Pre-diabetic	934 (40.96)	956 (38.41)	803 (34.79)	756 (31.78)	698 (28.30)	
Diabetic	219 (9.61)	199 (8.00)	198 (8.58)	176 (7.40)	177 (7.18)	
FPG-defined glycemic status, *n* (%)						0.450
Normoglycemia	1,971 (86.45)	2,158 (86.70)	1,989 (86.18)	2,053 (86.30)	2,122 (86.05)	
Pre-diabetes	185 (8.11)	222 (8.92)	206 (8.93)	226 (9.50)	231 (9.37)	
Diabetes	124 (5.44)	109 (4.38)	113 (4.90)	100 (4.20)	113 (4.58)	
**Disease rate**, ***n*** **(%)**
Hyperlipidemia	785 (34.43)	985 (39.57)	977 (42.33)	1,099 (46.20)	1,267 (51.38)	<0.001
Anemia	204 (8.95)	100 (4.02)	90 (3.90)	65 (2.73)	56 (2.27)	<0.001
Hepatic disease	28 (1.23)	17 (0.68)	20 (0.87)	26 (1.09)	41 (1.66)	0.014
Renal insufficiency	25 (1.10)	6 (0.24)	3 (0.13)	3 (0.13)	3 (0.12)	<0.001
Hyperuricemia	223 (9.78)	251 (10.08)	285 (12.35)	326 (13.70)	431 (17.48)	<0.001

*Data are presented as the mean ± standard deviation or Median (IQR: Q1–Q3) for continuous variables and percentage for categorical variables*.

* *The One-way ANOVA or Kruskal Wallis rank sum test or chi-square test was used for comparisons between subgroups. Q1–5, serum albumin level quintiles*.

### Univariate and Multivariate Analyses of HbA1c With Serum Albumin Levels

Univariate analyses were performed to analyze the associations between HbA1c and each anthropometrical and biochemical variable (Supplementary Table 1). Significant associations (all *P* < 0.001) were found between HbA1c and variables other than MCH, MCHC, and TP, and creatinine. Among them, a significant negative association was found between HbA1c and albumin (*P* < 0.0001).

Multivariate analyses were performed to measure the association between serum albumin and HbA1c. Both non-adjusted and adjusted models were analyzed to assess confounding. The association of albumin with HbA1c was measured with serum albumin levels treated as both continuous variables scaled to 2 g/L increments, and categorical variables by quintiles (Table 3). In the linear modeling, a per 2 g/L increment in albumin was associated with a lower HbA1c level [β: −0.06, 95% confidence interval (CI): −0.08 to −0.05, *P* < 0.0001] before adjusting for confounders (Model 1). In the sensitivity analyses, the negative association remained present after additional adjustment for potential confounders. After adjusting for all possible confounders (Model 4), HbA1c level was still independently inversely associated with albumin; for each 2 g/L increase in albumin, the HbA1c level decreased by 0.05% (β: −0.05, 95% CI: −0.06 to −0.04, *P* < 0.0001).

**Table 3:** Multivariate analysis of the association of serum albumin levels with HbA1c.

	**Model 1**^****a****^		**Model 2**^****b****^		**Model 3**^****c****^		**Model 4**^****d****^	
	**β (95% CI)**	***P***	**β (95% CI)**	***P***	**β (95% CI)**	***P***	**β (95% CI)**	***P***
Albumin, g/L^e^	−0.06 (−0.08, −0.05)	<0.0001	−0.02 (−0.03, −0.00)	0.0139	−0.02 (−0.04, −0.01)	0.0008	−0.05 (−0.06, −0.04)	<0.0001
Q1 (22.7–41.7 g/L)	0		0		0		0	
Q2 (41.7–43.1 g/L)	−0.08 (−0.13, −0.03)	0.0019	−0.03 (−0.07, 0.02)	0.2791	−0.03 (−0.07, 0.02)	0.2562	−0.06 (−0.08, −0.03)	<0.0001
Q3 (43.1–44.2 g/L)	−0.08 (−0.13, −0.03)	0.0014	−0.01 (−0.06, 0.03)	0.6005	−0.02 (−0.07, 0.03)	0.4352	−0.08 (−0.11, −0.05)	<0.0001
Q4 (44.2–45.5 g/L)	−0.14 (−0.19, −0.09)	<0.0001	−0.04 (−0.09, 0.01)	0.0916	−0.05 (−0.10, −0.00)	0.0452	−0.12 (−0.15, −0.09)	<0.0001
Q5 (45.5–53.4 g/L)	−0.19 (−0.24, −0.14)	<0.0001	−0.03 (−0.07, 0.02)	0.3223	−0.05 (−0.10, −0.00)	0.0384	−0.15 (−0.18, −0.12)	<0.0001
*P* trend	<0.001		0.254		0.027		<0.001	

*Model 1: ^a^Unadjusted; Model 2: ^b^adjusted for gender and age; Model 3: ^c^adjusted for gender, age, BMI, pulse, SBP and DBP; Model 4: ^d^adjusted for gender, age, BMI, pulse, SBP, DBP, FPG, TG, HDL-C, LDL-C, RBC, hematocrit, MCH, MCHC, ALT, AST, TP, DBIL, creatinine, BUN, and UA. ^e^per 2 g/L increment; Q1–5, serum albumin level quintiles*.

The multivariate regression analysis also showed that HbA1c levels were significantly lower for every increase in albumin level of a quintile (Table 3). In the analyses adjusted for all confounders (Model 4), HbA1c decreased by 0.15% in the fifth quintile compared with the first quintile (β: −0.15, 95% CI: −0.18 to −0.12, *P* < 0.0001).

### Effects of Other Variables on the Association Between HbA1c and Albumin

A subgroup analysis was used to explore the potential effects of other variables on the association between HbA1c and albumin (Supplementary Table 2). Stratified and interaction analyses were performed by gender, age, BMI, FPG, and several comorbidities. The results showed that the negative association between HbA1c and albumin was relatively stable in people of different genders or BMI, or people with or without hyperlipidemia or hyperuricemia.

However, differences were observed according to age, FPG, or anemia in the association between serum albumin levels and HbA1c. Specifically, the negative correlation was stronger in those people older than 45 years, with higher FPG, or with anemia (*P* for interaction = 0.0008, <0.001, and 0.0015, respectively). It is worth noting that FPG had the most significant effect on the association between HbA1c and albumin among these variables. The negative association was more significant in those people with a diabetes diagnosis based on FPG, with the HbA1c level lower by 0.31% for each 2 g/L increase in albumin (β: −0.31, 95% CI: −0.35 to −0.27, *P* < 0.0001).

### Analysis of the Non-linear Association Between Serum Albumin Levels and HbA1c Using Smooth Curve Fitting

Since the proportion of people with glycemic status as defined by HbA1c was different from that of people defined by FPG (Table 2), an investigation was conducted to determine differences in the negative associations among populations with different glycemic status defined by HbA1c.

**Figure 1:**
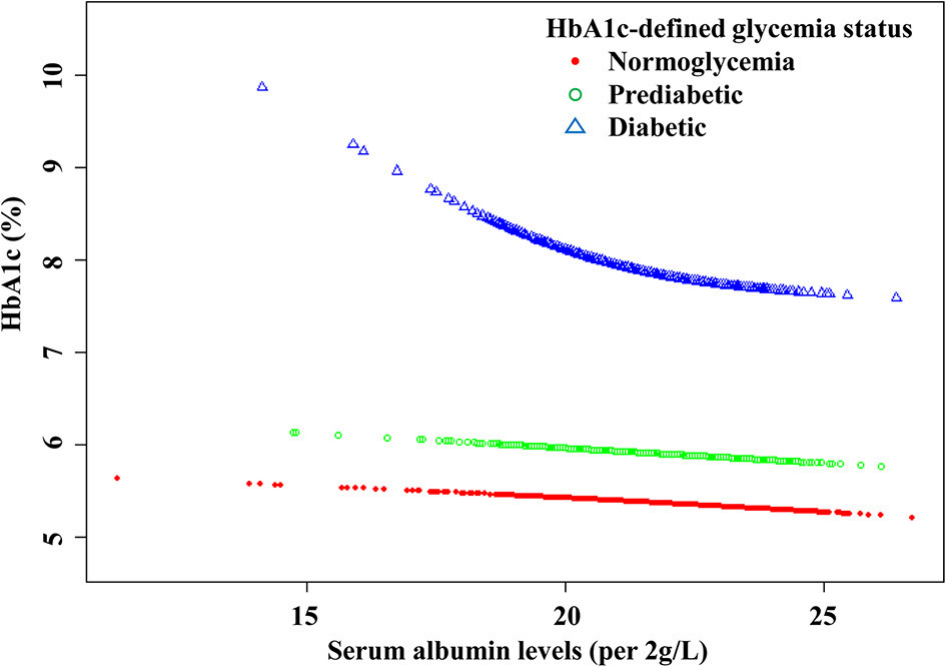
The curved lines illustrate the association between the serum albumin levels and HbA1c. A threshold, nonlinear association between serum albumin levels (per 2 g/L increment) and HbA1c was found (P < 0.0001) using a generalized additive model. The model was adjusted for gender, age, BMI, pulse, SBP, DBP, TG, HDL-CH, LDL-CH, FPG, RBC, HCT, MCH, MCHC, ALT, AST, TP, DBIL, creatinine, BUN, and UA.

There were linear but very weak negative associations between albumin levels and HbA1c in both normoglycemic (β: −0.01, 95% CI: −0.02 to −0.01, *P* < 0.0001) and pre-diabetic populations (β: −0.01, 95% CI: −0.02 to 0.00, *P* = 0.0533) defined by HbA1c (Figure 1; Table 4). The negative association between albumin and HbA1c was significantly stronger in the diabetic population defined by HbA1c. More importantly, a threshold, non-linear association between albumin and HbA1c was found using a generalized additive model (*P* = 0.0001; Figure 1).

**Table 4:** Analysis of the threshold effect of albumin on HbA1c using piecewise linear regression.

**Inflection point of albumin (per 2 g/L increment)**	**Effect size (β)**	**95% CI**	***P***
HbA1c-defined normoglycemia	−0.01	(−0.02, −0.01)	<0.0001
HbA1c-defined pre-diabetes	−0.01	(−0.01, 0.00)	0.0533
**HbA1c-defined diabetes**			
≥41.4	−0.10	(−0.17, −0.02)	0.0089
<41.4	−0.31	(−0.45, −0.17)	<0.0001

*The effect size of association was quantified by β and 95% CI. Adjusted for gender, age, BMI, pulse, SBP, DBP, TG, HDL-CH, LDL-CH, FPG, RBC, hematocrit, MCH, MCHC, ALT, AST, TP, DBIL, creatinine, BUN, UA*.

Threshold saturation effects were analyzed using multivariate piecewise linear regression based on the curve in Table 4. The data indicated that the turning point was 41.4 g/L. Specifically, HbA1c decreased with the elevation of albumin when the albumin level was ≥41.4 g/L (β: −0.10, 95% CI: −0.17 to −0.02, *P* < 0.0001). However, when the albumin level was below the inflection point, the negative association was significantly stronger: each 2 g/L increment in serum albumin level was significantly associated with a reduction by 0.31% in HbA1c (β: −0.31, 95% CI: −0.45 to −0.17, *P* < 0.0001).

### Analysis the Effect of Serum Albumin Levels on HbA1c/FPG-Defined Glycemic Status

The above results showed that glycemic status defined by HbA1c was significantly affected by the albumin level. A further comparative analysis was conducted of the proportion of people with HbA1c-defined glycemic status in each albumin interval.

**Figure 2:**
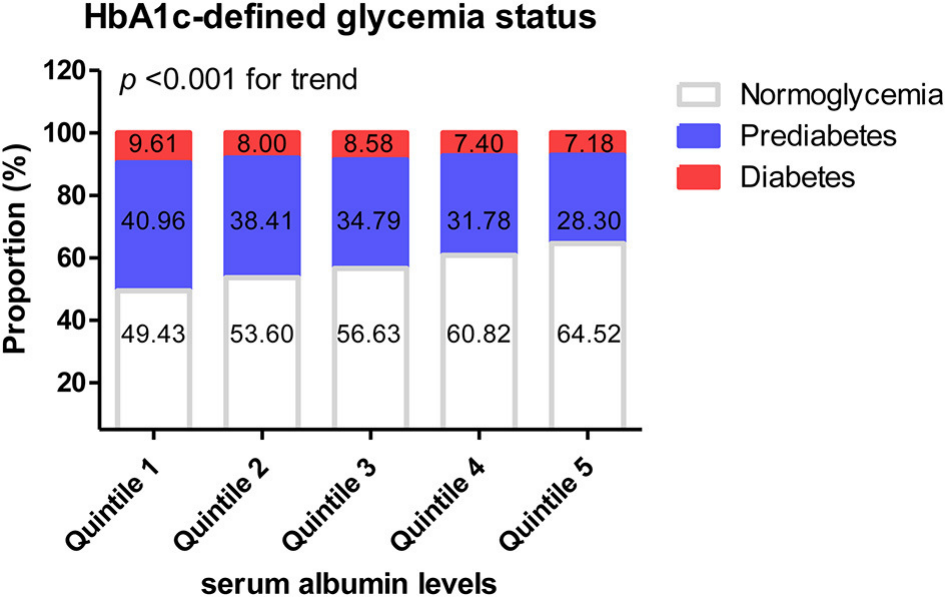
Quintiles of serum albumin levels and HbA1c-defined glycemic status.

The proportion of normoglycemic people (HbA1c <5.7%) in the highest-albumin interval was significantly higher than that in the lowest-albumin interval (64.52% cf. 49.43%, *P* < 0.001). The proportion of pre-diabetic people (HbA1c: 5.7–6.4%) in the lowest albumin interval was significantly higher than that in the highest (40.96% cf. 28.30%, *P* < 0.001). As albumin increased, the proportion of people with normoglycemia significantly increased, while both the proportion of people with pre-diabetes and diabetes decreased (*P* < 0.001 for the trend; Figure 2). However, when glycemic status was defined by FPG, there were no significant differences in the rates of normoglycemia, pre-diabetes, and diabetes among the various albumin intervals (Supplementary Figure 3).

An analysis was conducted of the influence of albumin levels on the diagnosis of glycemic status or glycemic management goals defined by various HbA1c thresholds. Table 5 shows odds ratios per 2 g/L increase in albumin, for HbA1c equal to or exceeding the thresholds of 5.7, 6.5, 7, and 8%. After adjustment for possible confounders, the odds of exceeding these thresholds decreased by 12–36% for each 2 g/L increase in albumin.

**Table 5:** Odds ratios (per 2 g/L increase in albumin) for exceeding various HbA1c cutpoints.

**Exposure**	**HbA1c** **≥** **5.7%**		**HbA1c** **≥** **6.5%**		**HbA1c** **≥** **7%**		**HbA1c** **≥** **8%**	
	**OR (95%CI)**	***p***	**OR (95%CI)**	***p***	**OR (95%CI)**	***p***	**OR (95%CI)**	***p***
Model 1	0.82 (0.80, 0.85)	<0.0001	0.90 (0.85, 0.95)	<0.0001	0.86 (0.81, 0.92)	<0.0001	0.79 (0.73, 0.87)	<0.0001
Model 2	0.94 (0.91, 0.98)	0.0050	0.90 (0.82, 0.99)	0.0247	0.81 (0.72, 0.92)	0.0011	0.68 (0.58, 0.79)	<0.0001
Model 3	0.95 (0.91, 0.99)	0.0090	0.90 (0.82, 0.99)	0.0290	0.81 (0.71, 0.92)	0.0011	0.67 (0.57, 0.78)	<0.0001
Model 4	0.88 (0.84, 0.93)	<0.0001	0.79 (0.73, 0.87)	0.0086	0.77 (0.67, 0.89)	0.0004	0.64 (0.53, 0.76)	<0.0001

*ORs (odds ratios) are given per 2 g/L increase in albumin with 95% CIs. Model 1 was unadjusted; Model 2 was adjusted for gender and age; Model 3 was adjusted for gender, age, BMI, Pulse, SBP and DBP; Model 4 was adjusted for gender, age, BMI, pulse, SBP, DBP, TG, HDL-C, LDL-C, RBC, hematocrit, MCH, MCHC, ALT, AST, TP, DBIL, creatinine, BUN, UA*.

## Discussion

In this large retrospective study, a significant negative association was found between HbA1c and albumin in all the participants. Importantly, in the HbA1c-defined diabetic population the negative association between HbA1c and albumin was significantly stronger and curved with a threshold. In addition, the classification of dysglycemia based on HbA1c was more sensitive to the albumin levels. Moreover, serum albumin levels significantly affected the odds ratios of the HbA1c thresholds that are commonly employed for diagnosis and management of diabetes.

Human serum albumin is the most abundant protein in the circulatory system, and a heavily glycosylated protein due to its abundance, characteristically with a relatively longer half-life (21 days) and greater number of lysine and arginine residues (21, 22). Thus, any variation in albumin levels may change the stoichiometry of plasma protein glycation, including hemoglobin, fibrinogen, and apolipoprotein (19, 23). Bhonsle et al. (19) and Rodriguez-Segade et al. (17) found that HbA1c levels negatively correlated with serum albumin levels due to competitive glycation inhibition by albumin. In accord with this, the present study determined that HbA1c decreased as serum albumin increased, and the mean HbA1c in the lowest albumin-level group was significantly higher than that of the group with the highest albumin level. Notably, participants enrolled in our study were not limited to diabetic participants but also included those with pre-diabetes or were normoglycemic, and the negative correlation between HbA1c and albumin was shown in all groups.

Several reports have suggested that alteration of HbA1c levels can be affected by several common factors, including erythrocyte turnover, iron deficiency, and kidney failure (24–26). In the present study, the negative association was maintained after making an allowance for all possible confounders. *In vivo*, albumin glycation precedes hemoglobin glycation because albumin is the most abundant protein in circulation with a large number of free lysine or arginine residues accessible for glycation (18, 22). This may reasonably explain why albumin could competitively protect hemoglobin glycation. In addition, changes in HbA1c concentration over the entire range of albumin concentrations may be a physiological rather than a pathological association, since the negative association between HbA1c and albumin was not affected by iron deficiency, or poor vascular or renal permeability to albumin (17, 27). However, with the change of albumin levels from the first to fifth albumin quintile, mean HbA1c value ranged across the threshold of HbA1c-defined pre-diabetes and the difference in proportion of HbA1c-defined pre-diabetic people was up to 12%, which demonstrated that albumin levels interfere with HbA1c-defined discrimination between pre-diabetes and normoglycemia in all participants.

In the present study, it was also observed that the negative correlation between HbA1c and albumin was greater in the presence of several common conditions, including older age (>45 y), higher FPG levels, and anemia. In patients older than 45 years or with anemia, a number of factors can contribute to lower HbA1c levels, including declined nutrient metabolism, blunted erythropoietin response, and altered erythrocyte life span (28, 29). When stratified and interaction analyses were performed by FPG, it was found that the negative association was significantly stronger with increasing FPG levels. This may be explained by the following. In patients with higher FPG levels, a large quantity of glycosylated albumin accumulates because albumin glycation precedes hemoglobin glycation (18). This leads to the modification of lysine residues of albumin, which weakens the ability of albumin to protect hemoglobin from glycosylation. Glycosylated albumin can also increase the HbA1c level by interacting with the receptor for advanced glycation end products (30). Moreover, given that albumin is characterized by easy aggregation and change in structure, there is a great chance of modification and polymerization in people with diabetes or other diseases (21, 22). With changes in albumin level, its physiological roles, such as the ability to protect hemoglobin from glycosylation, are affected (30, 31).

In the present study, since the negative effect of albumin on HbA1c was most significant in the FPG-defined diabetic population, we explored whether this phenomenon also exists in people with glycemic status defined by HbA1c. It was found that the association between albumin and HbA1c was strongest in HbA1c-defined diabetes, with a curve and threshold, and the negative correlation was significantly stronger at levels below 41.4 g/L. Given that the inflection point (41.4 g/L) is just around the lower limit of the normal range for albumin (40–55 g/L), these results indicate that patients with clinical hypoalbuminemia may have slightly higher HbA1c levels than those with normal albumin levels. It was also determined that the proportion of people with diabetes and pre-diabetes as defined by HbA1c decreased as albumin level increased, while the proportion of people with normoglycemia as defined by HbA1c changed inversely. Thus, it states that there may be a discrepancy when HbA1c is used to define the glycemic status of people with low albumin levels.

Although oral glucose tolerance test (OGTT) is often considered a gold standard for diagnosing diabetes and is the best criteria to define pre-diabetes, HbA1c was a more appropriate criterion than FPG when OGTT data was not available (32). HbA1c has been used clinically as a marker for the diagnosis and management of dysglycemia for a long time. In consideration of the importance of HbA1c for management of glycemia and control (33, 34), the effect of albumin on the odds ratio of different thresholds of HbA1c was analyzed in the current study. It was found that the odds of HbA1c equal to or exceeding the thresholds (i.e., 5.7, 6.5, 7, and 8%) decreased to varying degrees for each 2 g/L increase in albumin. These results show that albumin, especially low albumin levels, may affect the diagnosis and management of HbA1c-defined diabetes.

Some highlights of this study are worth mentioning. The minimum number of participants is 1,034 by sample size estimation, thus the number of participants was statistically sufficient in this study. The major anthropometric data and conventional biochemical and hematological parameters were collected and analyzed via multiple regression and sensitivity analysis, which ensured the reliability of the conclusion. The participants were then stratified by various conditions (e.g., age or anemia), and the possible influence of diseases on the conclusions were effectively controlled. Moreover, the finding of a curved association between HbA1c and albumin in HbA1c-defined diabetic participants, and the discovery of the inflection point (41.4 g/L), can be expected to provide a theoretical basis for clinical application.

Several shortcomings of this study should be considered. First, given the limitations of a retrospective study, it is possible that potential interfering factors were not entirely controlled. We cannot draw a causal conclusion regarding the inverse association between HbA1c and serum albumin. Second, the criteria used for diabetes diagnosis in the present study may not be precise; the FPG was only measured once and OGTT would not have been obtained in each participant undergoing routine physical examination as easily. Third, this population in southern China was in a single-center, and thus the conclusions may not be fully applicable to other regions or countries.

## Conclusions

Three conclusions may be drawn from this study regarding the influence of serum albumin on HbA1c level and HbA1c-defined glycemic status. Firstly, HbA1c increased with decreasing serum albumin levels. Secondly, the negative association between HbA1c and albumin has a threshold effect and the association was stronger in the HbA1c-defined diabetic population with hypoalbuminemia. Finally, a lower serum albumin level was associated with increased risk of an incorrect HbA1c-based identification of glycemic status and management. Although causality has not been demonstrated, these results in the present study indicate that serum albumin levels should be taken into account when interpreting HbA1c levels in clinical practice.

## Data Availability Statement

The raw data supporting the conclusions of this article will be made available by the authors, without undue reservation.

## Ethics Statement

The studies involving human participants were reviewed and approved by the ethics committee of Second Xiangya Hospital of Central South University. Written informed consent for participation was not required for this study in accordance with the national legislation and the institutional requirements.

## Author Contributions

XF and HT: conception, data analysis, and manuscript draft writing. XF, YY, SZ, and YFa: data collection. YD, YFu, QH, and QY: data verification. HT and LT: manuscript editing and supervision. All authors have accepted responsibility for the entire content of this submitted manuscript and approved submission.

## Conflict of Interest

The authors declare that the research was conducted in the absence of any commercial or financial relationships that could be construed as a potential conflict of interest.

## Supplementary Material

Supplementary Figure 1 Flow chart of the study population.

Supplementary Figure 2 Quintiles of serum albumin levels and HbA1c value. Quintiles of serum albumin levels and HbA1c in total participants.

Supplementary Figure 3 Quintiles of serum albumin levels and FPG-defined glycemia status.

Supplementary Table 1 Univariate analysis of characteristics of the participants with HbA1c.

Supplementary Table 2 Effect size of albumin (per 2 g/L increment) on HbA1c in the planned and exploratory subgroups.
