# Supplementary figures and images for: Influence of Serum Albumin on HbA1c and HbA1c-Defined Glycemic Status: A Retrospective Study
Source: Front Med (Lausanne). 2021 May 12;8:583093. doi: 10.3389/fmed.2021.583093 (PMC8149759; doi:10.3389/fmed.2021.583093)

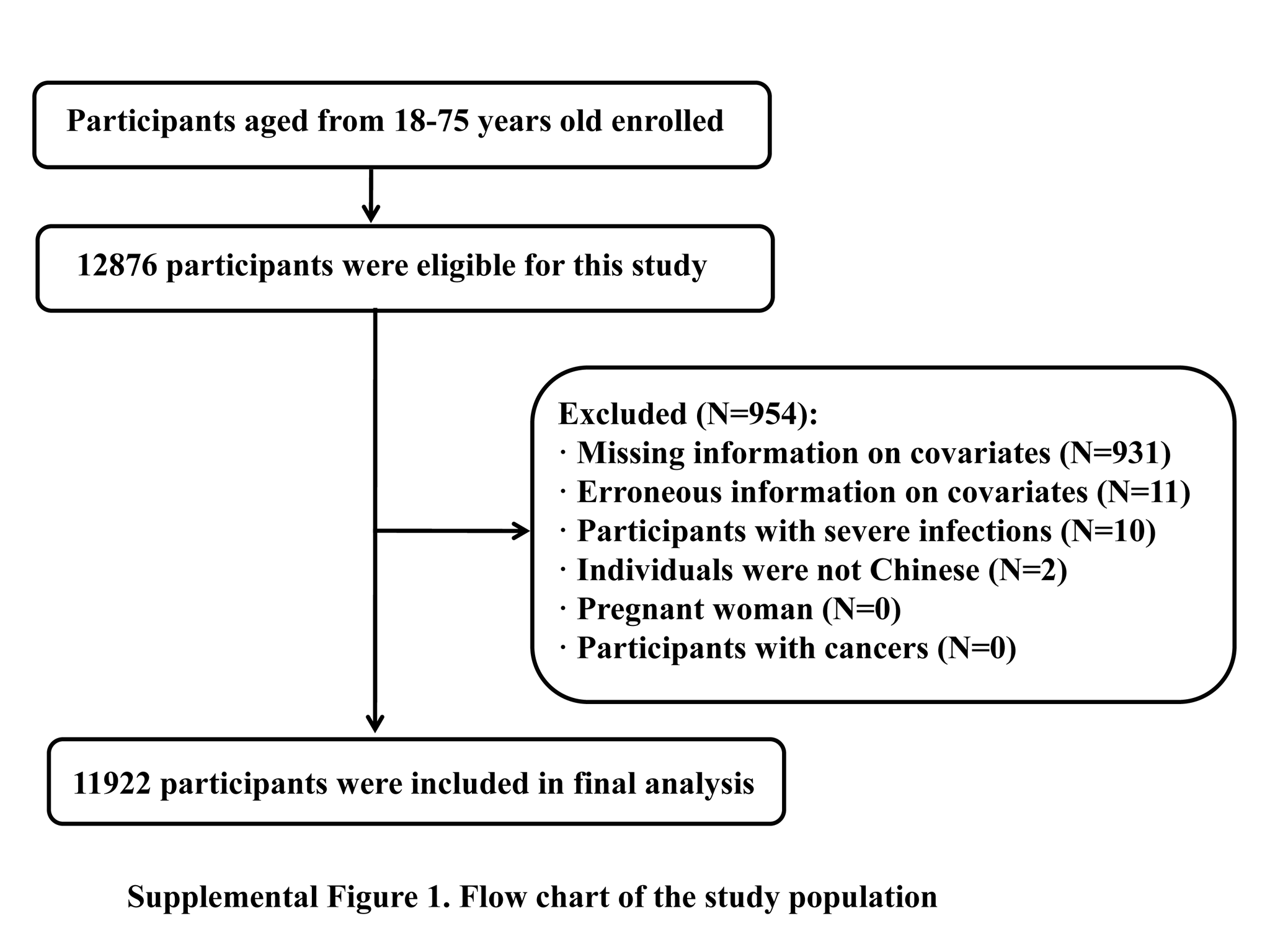

Supplement: Supplementary Figure 1 — Flow chart of the study population. [file Image_1.TIF]

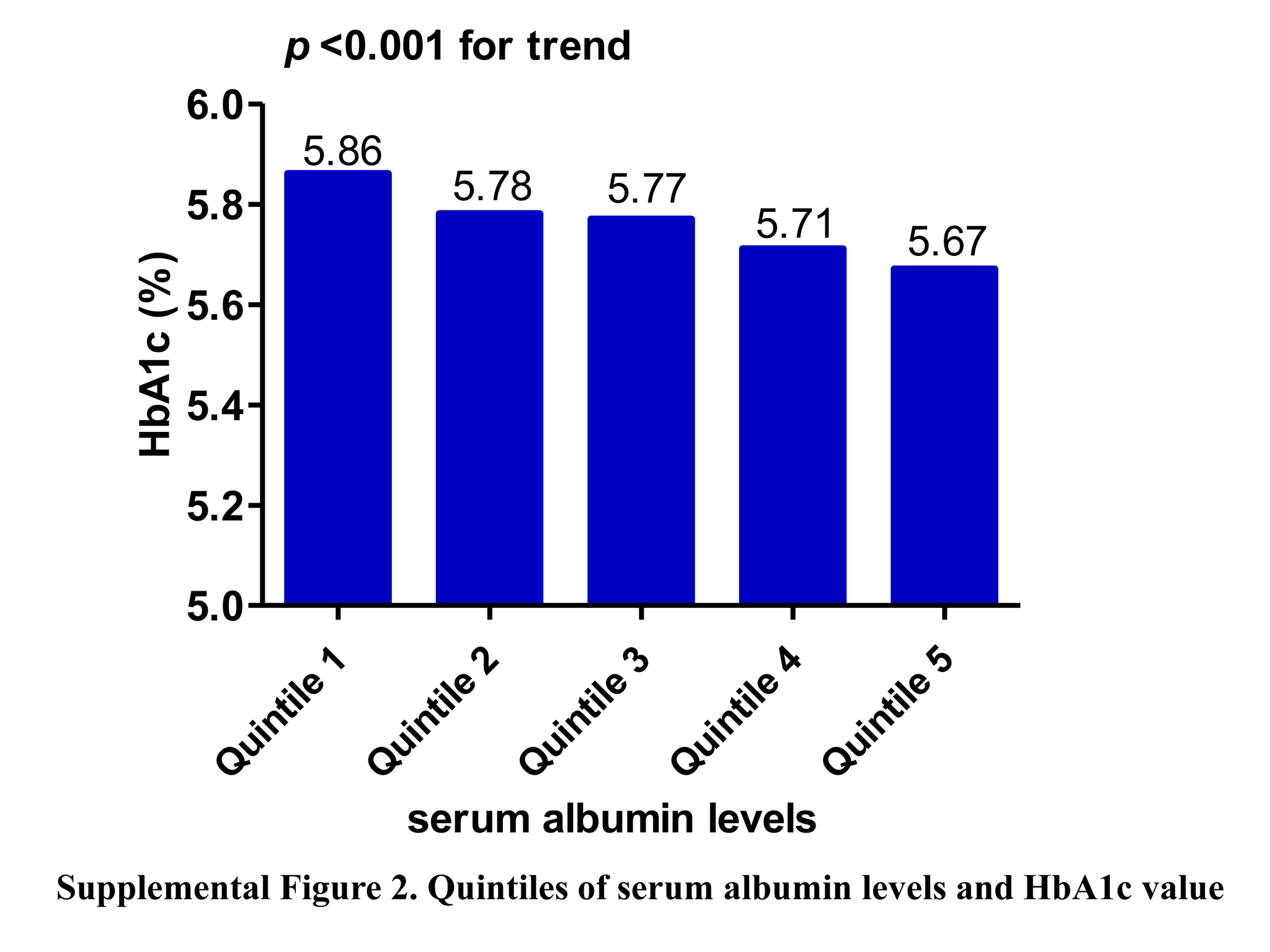

Supplement: Supplementary Figure 2 — Quintiles of serum albumin levels and HbA1c value. Quintiles of serum albumin levels and HbA1c in total participants. [file Image_2.TIF]

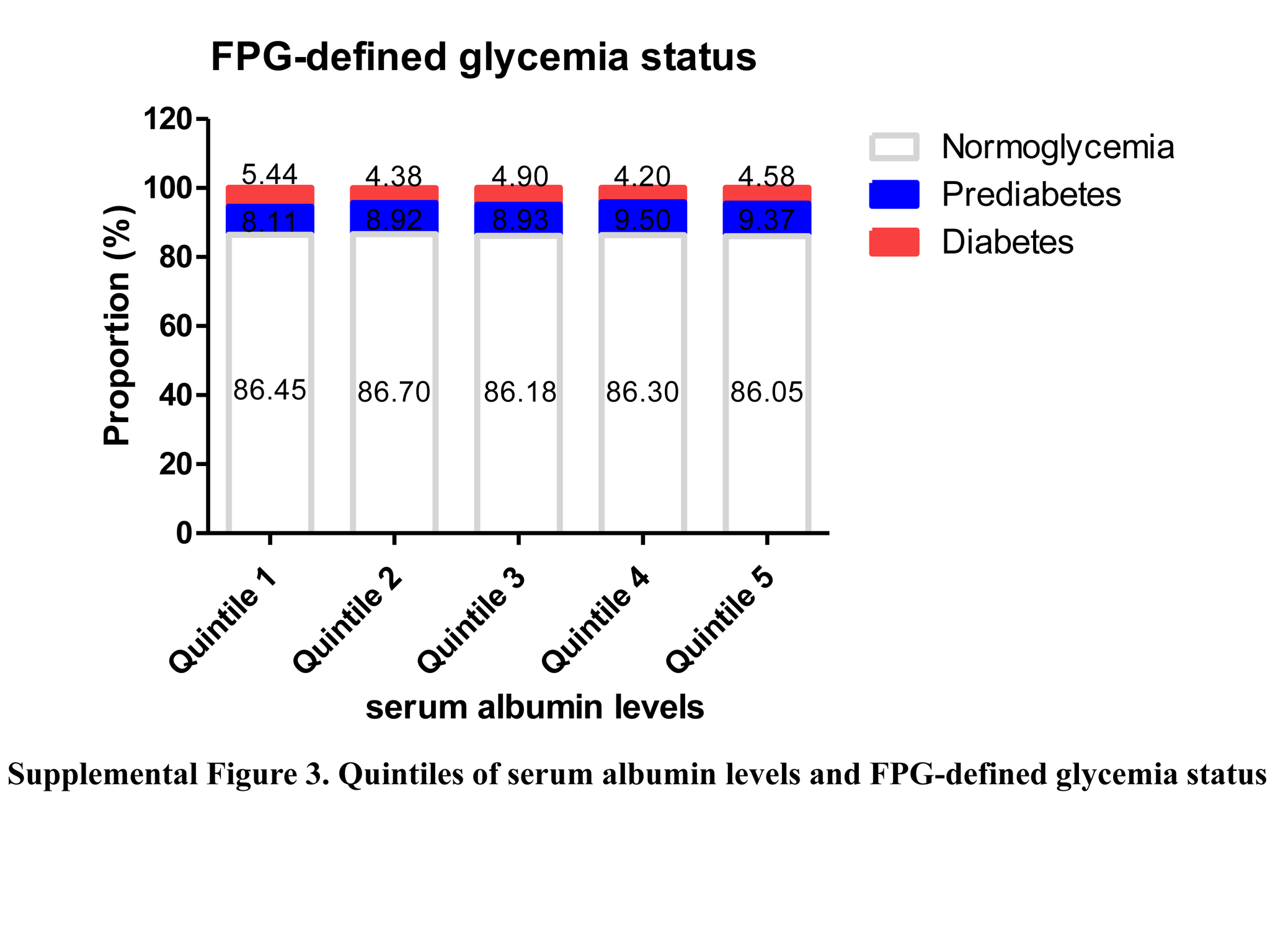

Supplement: Supplementary Figure 3 — Quintiles of serum albumin levels and FPG-defined glycemia status. [file Image_3.TIF]
