# Supplementary material for: Influence of Serum Albumin on HbA1c and HbA1c-Defined Glycemic Status: A Retrospective Study
Source: Front Med (Lausanne). 2021 May 12;8:583093. doi: 10.3389/fmed.2021.583093 (PMC8149759; doi:10.3389/fmed.2021.583093)
Supplement: Supplementary Table 1 — Univariate analysis of characteristics of the participants with HbA1c. [file Table_1.DOCX]

**Supplemental Tables**

**Supplemental Table 1 Univariate analysis of characteristics of the participants with HbA1c**

| **Variable** | **β (95% CI)** | ***p*** |
| --- | --- | --- |
| Age | 0.02 (0.02, 0.02) | <0.0001 |
| Gender |  |  |
| Female | 0 |  |
| Male | 0.21 (0.18, 0.24) | <0.0001 |
| BMI | 0.05 (0.04, 0.05) | <0.0001 |
| Pulse | 0.01 (0.00, 0.01) | <0.0001 |
| SBP | 0.01 (0.01, 0.01) | <0.0001 |
| DBP | 0.01 (0.01, 0.01) | <0.0001 |
| FPG | 0.50 (0.49, 0.51) | <0.0001 |
| RBC | 0.19 (0.16, 0.22) | <0.0001 |
| Hemoglobin | 0.00 (0.00, 0.01) | <0.0001 |
| Hematocrit | 0.02 (0.02, 0.02) | <0.0001 |
| MCV | -0.01 (-0.01, -0.00) | <0.0001 |
| MCH | -0.01 (-0.02, -0.00) | 0.0035 |
| MCHC | 0.00 (0.00, 0.00) | 0.0399 |
| ALT | 0.00 (0.00, 0.01) | <0.0001 |
| AST | 0.00 (0.00, 0.00) | <0.0001 |
| Albumin | -0.06 (-0.08, -0.05) | <0.0001 |
| TP | 0.00 (0.00, 0.01) | 0.0178 |
| DBIL | -0.03 (-0.04, -0.02) | <0.0001 |
| TC | 0.13 (0.11, 0.14) | <0.0001 |
| TG | 0.09 (0.08, 0.10) | <0.0001 |
| HDL-C | -0.35 (-0.40, -0.30) | <0.0001 |
| LDL-C | 0.15 (0.13, 0.17) | <0.0001 |
| Creatinine | 0.00 (0.00, 0.00) | 0.0022 |
| BUN | 0.07 (0.06, 0.08) | <0.0001 |
| UA | 0.00 (0.00, 0.00) | <0.0001 |

**Note:** β are given per year increase in age, for male vs. female, per 2 g/L in albumin, per 1 unit in the other parameters with 95% CIs.

**Supplemental Table 2 Effect size of albumin (per 2g/L increment) on HbA1c in the planned and exploratory subgroups.**

| **Characteristic** | **No. of participants** | **Effect size (95% CI)** | ***p*** | ***p* for interaction** |
| --- | --- | --- | --- | --- |
| **Gender** |  |  |  | 0.1899 |
| Male | 6044 | -0.06 (-0.07, -0.05) | <0.0001 |  |
| Female | 5878 | -0.05 (-0.06, -0.04) | <0.0001 |  |
| **Age, y** |  |  |  | 0.0008 |
| ≤45 | 4496 | -0.04 (-0.06, -0.03) | <0.0001 |  |
| >45 | 7426 | -0.07 (-0.09, -0.06) | <0.0001 |  |
| **BMI, kg/m^2^** |  |  |  | 0.8252 |
| <25 | 7286 | -0.05 (-0.06, -0.04) | <0.0001 |  |
| 25–28 | 3195 | -0.06 (-0.08, -0.04) | <0.0001 |  |
| ≥ 28 | 1441 | -0.05 (-0.08, -0.03) | <0.0001 |  |
| **FPG, mmol/L** |  |  |  | <0.0001 |
| <5.6 | 10293 | -0.02 (-0.03, -0.01) | 0.0011 |  |
| 5.6~7.0 | 1070 | -0.05 (-0.08, -0.01) | 0.0045 |  |
| ≥7.0 | 559 | -0.31 (-0.35, -0.27) | <0.0001 |  |
| **Hyperlipemia** |  |  |  | 0.6627 |
| No | 6809 | -0.05 (-0.06, -0.04) | <0.0001 |  |
| Yes | 5113 | -0.06 (-0.07, -0.04) | <0.0001 |  |
| **Anemia** |  |  |  | 0.0015 |
| No | 11407 | -0.05 (-0.06, -0.04) | <0.0001 |  |
| Yes | 515 | -0.10 (-0.12, -0.07) | <0.0001 |  |
| **Liver disease** |  |  |  | 0.1536 |
| No | 11790 | -0.06 (-0.07, -0.05) | <0.0001 |  |
| Yes | 132 | -0.00 (-0.08, 0.08) | 0.9968 |  |
| **Kidney disease** |  |  |  | 0.2498 |
| No | 11882 | -0.05 (-0.06, -0.05) | <0.0001 |  |
| Yes | 40 | 0.03 (-0.11, 0.18) | 0.6781 |  |
| **Hyperuricemia** |  |  |  | 0.0987 |
| No | 10406 | -0.06 (-0.07, -0.05) | <0.0001 |  |
| Yes | 1516 | -0.04 (-0.06, -0.01) | <0.0001 |  |

**Notes:** The effect size of association was quantified by β and 95% CI. Adjusted for gender, age, BMI, pulse, SBP, DBP, RBC, hematocrit, MCH, MCHC, ALT, AST, TP, DBIL, TG, HDL.CH, LDL.CH, creatinine, BUN, UA, except the subgroup variable.
